# Corps étranger intraoculaire et endophtalmie: facteurs de risque et prise en charge

**DOI:** 10.11604/pamj.2019.33.258.18554

**Published:** 2019-07-26

**Authors:** Olfa Fekih, Mariem Touati, Hsouna Mehdi Zgolli, Sonya Mabrouk, Omar Haj Said, Imene Zeghal, Leila Nacef

**Affiliations:** 1 Institut d'Ophtalmologie Hedi Raies, Service A, Tunis, Tunisie

**Keywords:** Endophtalmie, corps étranger intraoculaire, traumatisme à globe ouvert, Endophthalmitis, intraocular foreign body, open globe injury

## Abstract

Le but est d'identifier les facteurs de risque de survenue d'endophtalmie post traumatique (EPT) avec corps étrangers intra oculaire (CEIO) dans les traumatismes pénétrants du globe et d'évaluer les résultats de la prise en charge thérapeutique. Il s'agit d'une étude descriptive rétrospective portant sur 60 patients hospitalisés pour plaie pénétrante avec corps étranger intraoculaire à l'institut Hédi Raies d'ophtalmologie de Tunis, sur une période de 10 ans. Nous avons inclus dans notre travail les patients présentant un traumatisme oculaire pénétrant avec CEIO présentant une EPT. Nous avons également identifié les facteurs de risque cliniques de survenue d'endophtalmie chez ces patients. Nous avons colligé 60 patients présentant un traumatisme pénétrant avec corps étranger intraoculaire. Dix de ces patients soit 16,66% ont présenté des manifestations cliniques d'endophtalmie. Tous les CEIO étaient localisés dans le segment postérieur. L'acuité visuelle (AV) moyenne initiale était de 2,26 en Log MAR. L'AV moyenne finale était de 2,18 en Log MAR. Nous avons trouvé un lien statiquement significatif entre la survenue d'une endophtalmie et les facteurs suivants: l'origine rurale p=0,021, le délai d'extraction du CEIO p=0,01, la localisation postérieure du CEIO p=0.012, la rupture capsulaire p=0,022, le décollement de rétine associé p<0,0001. L'identification des facteurs de risque de survenue d'EPT vont permettre de mieux adapter la prise en charge thérapeutique et préventive de cette complication à fin d'améliorer le pronostic et la qualité de vie des patients.

## Abstract (en)

This study aims to identify the risk factors for post-traumatic endophthalmitis (PTE) due to intraocular foreign body (IFB) in patients with penetrating injuries of the globe and to assess treatment outcomes. We conducted a descriptive, retrospective study of 60 patients hospitalized for penetrating wound due to intraocular foreign body at the Hedi Rais Institute of Ophthalmology, Tunis, over a period of 10 years. Our study involved the patients with penetrating ocular trauma due to intraocular foreign body associated with post-traumatic endophthalmitis. We also determined the clinical risk factors for endophthalmitis in these patients. We collected data from the medical records of 60 patients with penetrating injury due to intraocular foreign body. Clinical symptoms of endophthalmitis were reported in ten of these patients (16.66%). All IFBs were located in the posterior segment. Mean LogMAR visual acuity (VA) at baseline was 2.26. Final Mean LogMAR VA was 2.18. We found a statically significant correlation between the occurrence of endophthalmitis and the following factors: rural origin p=0.021, delays in removing IFB p=0.01, posterior location IFB p=0.012, capsule rupture p=0.022, associated retinal detachment p<0.0001. The identification of risk factors for post-traumatic endophthalmitis allow for better treatment adaptation and preventive measures of this complication to improve prognosis and quality of life.

## Introduction

L'endophtalmie post-traumatique (EPT) est une complication redoutable des traumatismes à globe ouvert. La présence d'un corps étranger intraoculaire (CEIO) est un facteur bien connu pour augmenter le risque de survenu de cette complication [1]. La prise en charge rapide du ces traumatismes avec suture de la plaie est la seule recommandation clairement établie. Les autres procédures curatives ou préventives ne sont pas encore bien codifiées. Les buts de notre étude étaient d'identifier les facteurs de risque de survenue d'endophtalmie post traumatique avec corps étrangers intra oculaire et d'évaluer les résultats de la prise en charge thérapeutique.

## Méthodes

Il s'agit d'une étude descriptive rétrospective portant sur 60 patients hospitalisés pour plaie pénétrante avec corps étranger intraoculaire au Service A de l'Institut Hédi Raies d'Ophtalmologie de Tunis, sur une période de 10 ans allant de 2007 à 2017. Nous avons inclus dans notre travail les patients présentant un traumatisme oculaire pénétrant avec corps étranger intraoculaire selon la définition établie par la classification de Birmingham Eye Trauma Terminology (BETT) [1] et présentant une EPT. Nous avons exclu de notre étude: les corps étrangers perforants, les corps étrangers cornéens superficiels non pénétrants, les patients avec plaies pénétrantes sans CEIO, les patients avec corps étrangers orbitaires extra-oculaires, les patients atteints d'une pathologie oculaire antérieure responsable d'une baisse de l'acuité visuelle. Nous avons recueilli les informations à partir des dossiers des malades, les données suivantes ont été précisées: le profil démographique, le délai de consultation, le mécanisme du traumatisme, la nature et la localisation du CEIO, l'acuité visuelle initiale, les résultats de culture de microorganisme, le délai d'extraction du CEIO, la méthode chirurgicale utilisée, les lésions associées, l'utilisation d´antibiotiques systémiques et intravitréens, les résultats anatomiques et fonctionnels finaux. Le suivi des patients variait de 6 mois à 10 ans avec un suivi moyen de 27,56 mois. Tous les patients ont bénéficié d'une suture en urgence de la plaie dès leur consultation.

Le diagnostic d'endophtalmie post traumatique a été évoqué sur la présence d'un ou plusieurs de ces signes: douleur, rougeur, baisse de l'acuité visuelle, sécrétions purulentes, chémosis, œdème cornéen, une réaction inflammatoire de la chambre antérieure avec tyndall, hypopion, tyndall vitréen, atteintes rétiniennes, présence d'échos intravitréennes à l'échographie en mode B réalisée après suture de la plaie. Tous les patients ont bénéficié d'injection intravitréenne (IVT): vancomycine (vancocine ^®^) 1 mg dans 0,1cc de sérum physiologique (NaCl 0,9%) et ceftazidime (fortum ^®^) 2 mg dans 0,1 cc de sérum physiologique (NaCl 0,9%). Tous les patients ont bénéficié d'une double antibiothérapie locale à base de collyre fortifiée et double antibiothérapie générale probabiliste débutée après réalisation de prélèvements endoculaires à visée microbiologique (prélèvement au niveau de la chambre antérieure et ou prélèvements vitréens): 1) dilution des collyres fortifiés: Vancomycine (50mg/kg) et ceftazidime (100mg/kg) par heure. 2) l'antibiothérapie systématique: une fluoroquinolone 750 mg deux fois par jour per os et imipenème (Tienam^®^) 500mg *4 par jour en injection intraveineuse. Cette antibiothérapie a été par la suite adaptée en fonction des résultats bactériologiques. Tous les patients ont bénéficié d'une vitrectomie (20 ou 23 gauges): cette vitrectomie a été réalisée afin de procéder à des prélèvements endoculaires, de libérer la cavité vitréenne et d'extraire le CEIO. Afin d'identifier les facteurs de risque cliniques associés à un risque significatif de survenue d´endophtalmie chez les patients victime d'une plaie pénétrante avec CEIO, les données ont été saisies au moyen du logiciel Excel et analysées en utilisant le logiciel SPSS version 24. La comparaison des moyennes sur séries indépendantes ont été effectuées au moyen de test F de Snedecor d'analyse de la variance paramétrique (ANOVA à un facteur). Les comparaisons des pourcentages ont été effectuées par le test du chi-deux de Person. En cas de non validité de ce test, ces comparaisons ont été faites par le test exact bilatéral de Fisher. Dans tous les tests statistiques, le seuil de signification a été fixé à 0,05.

## Résultats

Nous avons colligé 60 patients présentant un traumatisme pénétrant avec corps étranger intraoculaire. Dix de ces patients soit 16,66% ont présenté des manifestations cliniques d'endophtalmie: le Tableau 1 résume les caractéristiques démographiques et cliniques de ces dixpatients. L'âge moyen de ces patients était de 25,3 ans. Le délai de consultation était < 24 h dans 5 cas et > 24h dans les 5 autres. Neuf patients étaient d'origine rurale et un seul patient était d'origine urbaine. La survenue de l'endophtalmie était dès l'examen initial dans 3 cas et après suture de la plaie dans les 7 autres. Le mécanisme du traumatisme était le martèlement dans les 10 cas. L'origine du corps étranger était métallique dans tous les cas. La porte d'entrée était cornéenne dans 6 cas, cornéo-sclérale dans 2 cas, sclérale dans 2 cas. Tous les CEIO étaient localisés dans le segment postérieur. Cinq patients avaient une hémorragie intravitéenne, sept patients avaient un décollement de rétine associé. Sept patients avaient une rupture capsulaire associée à une cataracte post traumatique. L'acuité visuelle initiale (AVI) était inférieure à 1/10 dans 9 cas et supérieure à 1/10 dans un seul cas. L'acuité visuelle initiale moyenne était de 2,2>6 en Log MAR. La culture était positive dans 6 cas soit 60% des cas. Les Gram positifs étaient les organismes les plus isolés dans notre étude dans 5 cas soit 83,33% des cas (Tableau 1). Une vitrectomie à la pars plana 20 gauges a été réalisée pour un patient et 23 gauges pour 9 patients avec élargissement de l'orifice de sclérotomieafin d'extraire le CEIO. La vitrectomie avec une IVT d'antibiothérapie peropératoire était réalisé d'emblée dans 6 cas.

**Tableau 1:** Caractéristiques épidémiologiques et cliniques des patients présentant une endophtalmie post-traumatique (EPT) avec corps étranger intraoculaire (CEIO)

Cas	Age / sexe	Délai de consultation	Siège plaie	Nature CEIO	Siège CEIO	Délai suture plaie	Délai d’extraction CEIO	Méthode d’extraction	culture	AVi	MAVC f
1	16/M	1j	scléral	Métallique	Postérieur rétine	1j	Pas d’extraction	Vitréctomie	Aeromonas hydrophila	PLBO	PLBO
2	48/M	1j	cornée	Métallique	Postérieur vitré	1j	20 j	Vitréctomie Aimant	Staphylocoque epidermis	PLMO	PL-
3	24/M	10 j	cornée	Métallique	Postérieur rétine	10 j	12 j	Vitrectomie Aimant	Staphylocoque epidermis	PLMO	PL-
4	20/M	2 j	cornée	Métallique	Postérieur rétine	2 j	5 j	Vitréctomie Pince	négative	PLBO	CLD à 1 m
5	37/M	4 j	Cornée	Métallique	Postérieur rétine	Plaie colmatée	Pas d’extraction	Vitéctomie	Staphylocoque Aureus	PLBO	CLD à 2 m
6	32/M	1 j	cornée	Métallique	Postérieur rétine	1j	9 j	Vitréctomie Pince	négative	2/10	2/10
7	26/M	15 j	cornée	Métallique	Postérieur rétine	Plaie colmatée	26 j	Vitréctomie Pince	négative	PLBO	PLBO
8	27/M	10 j	Cornéo- sclérale	Métallique	Postérieur Vitré	Plaie colmatée	15 J	Vitréctomie Pince	Staphylocoque epidermis	PLMO	PLBO
9	23/M	1 j	Cornéo- sclérale	Métallique	Postérieur Sclére	1j	1 j	Vitréctomie puis extraction par voie externe	Staphylocoque epidermis	PLBO	PLBO
10	21/M	1 j	scléral	Métallique	Postérieur macula	1 j	25 j	Vitréctomie pince	Négative	PLBO	PLBO

Quatre patients ont bénéficié de deux injections intravitréennes d'antibiothérapie suivies de vitrectomie. Le délai moyen d'extraction du corps étranger après survenu du traumatisme étaitde 10,71 jours: le CEIO était extrait dans un délai > 7 jours dans sept cas et dans un délai <7 jours dans deux cas. Dans deux cas le corps étranger n'a pas été extrait: dans le premier cas le CEIO était profondément enchâssé dans la paroi et dans le deuxième cas le CEIO était très périphérique. L'évolution sur le plan anatomique a été marquée par la phtyse du globe dans 2 cas. Ces 2 patients avaient un décollement de rétine associé à l'EPT. Les patients présentant une cataracte traumatique associée ont tous bénéficié d'une chirurgie combinée: vitrectomie et chirurgie de la cataracte. La meilleure acuité visuelle corrigée finale (MAVCf) était comme suit: perception lumineuse négative (PL-) dans 2 cas, perception lumineuse bien orientée (PLBO) dans 5 cas, compte les doigts (CLD) dans 2 cas, 2/10 dans 1 cas. La MAVCf moyenne était de 2,18 en Log MAR. Nous avons noté une dégradation de l'AV dans 50% des cas, une stabilisation dans 30% des cas et une amélioration de l'AV dans seulement 20% des cas. Nous avons établi un lien statiquement significatif entre la survenue d´une endophtalmie et les facteurs suivants: l'origine rurale: p=0,021, le délai d'extraction du CEIO: p=0,013, la localisation postérieure du CEIO: p= 0,012, la rupture capsulaire: p=0,022, un DR associé: p<0,001. Il n´y avait pas d'association statistiquement significative entre l´endophtalmie et: l'âge: p=0,792, le sexe: p=0,245, le délai entre le traumatisme et la suture de la plaie: p=0,215, la taille du CEIO: p=0,111, la localisation de la plaie: p=0,320.

## Discussion

Nous avons retrouvé dans notre étude un lien significatif entre la survenue d'endophtalmie post traumatique avec corps étranger intraoculaire et les facteurs suivants: l'origine rurale, le délai d'extraction du CEIO, la localisation postérieure du CEIO, La rupture capsulaire et le DR associé p<0,0001. En effet, tous les traumatismes avec CEIO peuvent se compliquer d'enophtalmie et comme pour notre étude certains éléments sont retrouvés comme facteurs favorisant cette complication redoutable. L'incidence dans les milieux ruraux est augmentée essentiellement par le fait que l'inoculum causé les agents vulnérants à l'origine de ces traumatismes est important augmentant ainsi le risque d'infection intraoculaire. De plus, les lésions rencontrées dans ces régions sont souvent plus étendues et plus délabrées. Les germes pourraient être également plus virulents et plus résistants aux antibiotiques notamment en cas d'agent vulnérant tellurique [2]. La prise en charge des traumatismes du globe et notamment des traumatismes à globes ouverts doit être réalisée en urgence dans les 24 heures. Au-delà de cette limite, le risque d'EPT est statistiquement plus important. Les dégâts proviennent non seulement des facteurs toxiques produits par les microorganismes, mais aussi de l´afflux de cellules inflammatoires [3]. Une étude rétrospective sur 492 yeux avec un traumatisme à globe ouvert avec CEIO retrouvait une incidence moyenne d'EPT de 6,9%. Ce taux était statistiquement plus important en cas de prise en charge chirurgicale initiale supérieure à 24 heures (3,5% vs 13,4%, p < 0,0001) [3]. L'ablation rapide du CEIO par vitrectomie dans les 24 heures permet également de diminuer rapidement l'inoculum et de retirer le vitré qui est le lieu de prolifération préférentiel des germes. Cette particularité ne serait pas confirmée en cas de CEIO balistiques du fait de la stérilisation des CEIO pendant leur trajet [4]. Dans une étude de 62 cas de traumatismes à globes ouverts avec CEIO du segment postérieur, les patients ont été divisés en deux groupes selon que le CEIO était enlevé avant en même temps que la suture de la plaie ou en différé [5]. Une endophtalmie a été notée dans 15,7% des cas dans le groupe intervention différée versus 2,3% dans l'autre groupe avec une différence statistiquement significative (p= 0,046). Nous avons trouvé dans notre étude une association significative entre le retard d'extraction du CEIO et la survenue d'endophtalmie (p=0,022). En revanche, Woodcock et ses collègues n´ont pas trouvé d'association significative entre le retrait tardif du CEIO et le risque d´endophtalmie [6]: leurs données ont démontré un risque accru d´endophtalmie si ni antibiotiques prophylactiques systémiques prophylactiques ou intravitréens n'ont pas été utilisés.

La majorité des CEIO responsables d'endophtalmie post traumatique sont localisés dans le segment postérieur d'après les données de la littérature [6]. Ces CEIO postérieurs ont un pronostic visuel plus réservé que les CEIO antérieurs puisqu'ils engendrent également des lésions rétiniennes surajoutées [3]. Dans notre étude les corps étrangers ayant engendré une endophtalmie étaient tous localisés dans le segment postérieur (p=0,012). L'atteinte cristallinienne est un facteur de risque reporté par plusieurs études [2,5,7]. Plusieurs hypothèses sont avancées pour expliquer ce facteur de risque. Tout d'abord, la lésion cristallinienne donne mécaniquement aux micro-organismes un accès plus facile et rapide au vitré. Secondairement, les fragments cristalliniens pourraient être un substrat énergétique pour ces micro-organismes. Enfin, les lésions cristalliniennes entraînent des modifications de la sécrétion et la résorption de l'humeur aqueuse diminuant ainsi sa clairance et l'évacuation des germes qui la contaminent [5,8]. Dans notre série nous avons retrouvé une association significative entre la rupture capsulaire et la survenue d'endophtalmie (p=0,021). L'apparition d'un décollement de rétine au moment de l'infection est de pronostic sévère car il signe une atteinte initiale sévère ou une virulence particulière du germe. Dans une série, rapportée par Affeldt et ses collègues, tous les cas d'EPT avec décollement de la rétine ont développé phtyse du globe [9,10]. Le principal facteur de risque de phtyse est l'apparition d'une prolifération vitréo rétinienne. Celle-ci est présente dans 88% des cas de décollement de rétineau cours d'EPT [9,10]. Deux de nos patients présentant une endophtalmie avec un DR ont évolué vers la phtyse. Nous avons également retrouvé une association significative entre la présence de décollement de rétine et la survenue d'endophtalmie (p<0,0001). D'autres facteurs ont été retrouvés dans la littérature comme étant des facteurs de risque d'endophtalmie mais non retrouvés dans notre série. En effet, les patients âgés de plus de 50 ans auraient un risque infectieux supérieur et ceci peut s'expliquer par la diminution de la réponse immunitaire avec l'âge [4]. Dans notre série, l'âge n'était pas associé à la survenue d'EPT et ceci s'explique par le fait que 90% de nos patients étaient âgés de moins de 50 ans. La nature du CEIO est également un facteur identifié dans différentes études: les CEIO végétaux sont pourvoyeurs d'infections polymicrobiennes et fungiques dont la prise en chargereste délicate [2,11,12]. À l'inverse, à atteinte anatomique équivalente, les CEIO à haute vélocité (balistique, engins explosifs) ne sont pas très pourvoyeurs d'EPT car ils se stérilisent par la forte température qu'ils dégagent [8,13]. Dans notre série, les corps étrangers incriminés dans les traumatismes sont de nature métallique ce qui explique que ce facteur n'a pas été retrouvé comme facteur de risque. L'issu du vitré est également un facteur fréquemment incriminé dans la littérature: l'issu du vitré permet un accès direct au lieu de l'infection aux germes augmentant ainsi le taux d'EPT [14].

Il est important de bien identifier ces facteurs de risque puisque l'une des premières difficultés de la prise en charge des endophtalmies post traumatiques est de poser rapidement le diagnostic positif à fin d'instaurer un traitement adéquat. En effet, le diagnostic d'endophtalmie peut être difficile à poser dans le contexte d'inflammation post-traumatique puisque la majorité des signes cliniques est commune aux deux entités. De plus, malgré l'évolution des modalités de traitement médico-chirurgicale, le pronostic des endophtalmies post traumatiques avec CEIO reste réservé d'où l'importance d'un traitement préventif. Grace à l'identification de ces facteurs de risque, le praticien peut sélectionner les traumatismes avec CEIO à haut risque d'endophtalmie et adapter sa prise en charge en fonction de ce risque. L'antibioprophylaxie locale et systémique est recommandée par la majorité des auteurs notamment en présence de CEIO malgré l'absence de preuves scientifiques rigoureuses [6]. L'usage des fluoroquinolones est souvent recommandé par la plupart des auteurs car il s'agit d'une classe d'antibiotique ayant un large spectre d'action avec une bonne pénétration oculaire [15]. Concernant le traitement par voie systémique, la ciprofloxacine et plus récemment les fluoroquinolones de quatrième génération (moxifloxacime) présentent l'avantage d'une meilleure couverture des cocci Gram positif et des anaérobies. L'association vancomycine- ceftazidine est également largement utilisée puisqu'elle permet de couvrir la majorité du spectre de germe incriminés dans les endophtalmies post traumatiques notamment le *Bacillus cereus* et *Clostridium*, germes spécifiques des traumatismes oculaires avec CEIO telluriques [16].

Tous nos patients ont bénéficié d'une antibioprophylaxie locale et systémique. En cas de suspicion de mycose, notamment lors de la présence d'un CEIO végétal, le candida étant l'espèce la plus incriminée, il est recommandé de prescrire de l'Amphotéricine B par voie locale et le fluconazole par voie générale [17-19]. Le recours aux injections intravitréenes (IVT) prophylactique est sujet à controverse [17] contrairement aux IVT curatives. Néanmoins, il parait judicieux d'indiquer une IVT préventive d'antibiotiques en présence d'un ou plusieurs facteurs de risque d'endophtalmie puisque cette voie d'administration permet d'atteindre des concentrations très élevées localement. Les antibiotiques recommandés sont les même que pour le traitement curatif: céftazidime (2,25 mg/ml) et vancomycine (1 mg/0,1 ml). La gentamycine et l'amikacine sont moins utilisés à cause risque de toxicité rétinienne. En cas de suspicion de Bacillus, l'utilisation de la clindamycine peut être proposée [17]. En cas de risque d'endophtalmie fongique, une IVT d'amphotrecine B peut être indiquée. Dans notre série, 12% des patients ont bénéficié d'une IVT prophylactique en fin de suture ou après extraction du CEIO. Le taux d'endophtalmie dans notre série était de 16.6%. Ce taux peut atteindre 36% dans la littérature (Tableau 2) puisque la présence d'un corps étranger intraoculaire augmente significativement le taux d'endophtalmie en cas de traumatisme ouvert du globe. Concernant la prise en charge thérapeutique, en raison de la virulence des organismes impliqués dans les endophtalmies post traumatique et vu l'acuité visuelle initiale le plus souvent médiocre, une vitrectomie précoce serait appropriée pour la plupart des patients [20,21]. Nous avons indiqué une vitrectomie de première intention dans notre série dans 60% des cas. Des études à large échelles sont nécessaires, comme l'EVS pour l'endophtalmie postopératoire [22], pour codifier les indications de la vitrectomiede première intention en matière d'endophtalmie post traumatique.

**Tableau 2:** Fréquence d'endophtalmie post-traumatique (EPT) avec corps étranger intraoculaire (CEIO)

Auteurs	Année d’étude	Nombre de cas	Pourcentage (%)
Riteshkumar shah [19]	2017	4/11	36%
Ibraheem waheed Ademola [20]	2016	3/64	4,7%
Zafer O [21]	2015	9/58	15,5%
Simona Delia Nicoara [22]	2015	6/21	28,57%
Notre étude	2017	**10/60**	**16,66%**

## Conclusion

Les endophtalmies compliquant les traumatismes avec corps étranger intraoculaire garde un pronostic réservé malgré les avancées de la chirurgie vitréo rétinienne et l'avènement de nouvelles familles d'antibiotiques. L'identification des facteurs de risque de survenue de cette complication vont permettre de mieux adapter la prise en charge thérapeutique de cette pathologie à fin d'améliorer le pronostic et la qualité de vie des patients. Le rôle des mesures préventives notamment des IVT prophylactiques reste à déterminer mais semble être justifiable devant un risque élevé d'endophtalmie.

### État des connaissances actuelles sur le sujet

- Les traumatismes oculaires à globe ouvert sont hautement associés à la présence d'un corps étranger intra oculaire;
- L'endophtalmie liée à ce type de traumatisme est fréquente et présente un problème de santé publique majeure.

### Contribution de notre étude à la connaissance

- Cerner les différents facteurs de risque des endophtalmie liées aux corps étranger intraoculaire;
- Proposer une attitude thérapeutique adaptée selon les profils particuliers des patients du bassin méditerranéen.

## Conflits des intérêts

Les auteurs ne déclarent aucun conflit d'intérêts.

## References

[1] Kuhn F, Morris R, Witherspoon CD, Mester V (2004). La Birmingham Eye Trauma Terminology (BETT): un système de classification standardisé pour la traumatologie oculaire. J Fr Ophtalmol.

[2] Essex RW, Yi Q, Charles PGP, Allen PJ (2004). Post-traumatic endophthalmitis. Ophthalmology.

[3] Cebulla CM, Flynn HW (2009). Endophthalmitis after Open Globe Injuries. American journal of ophthalmology.

[4] Thompson WS, Rubsamen PE, Flynn HW, Schiffman J, Cousins SW (1995). Endophthalmitisafter penetrating trauma: risk factors and visual acuity outcomes. Ophthalmology.

[5] Jonas JB, Budde WM (1999). Early versus late removal of retained intraocular foreign bodies. Retina.

[6] Woodcock MG, Scott RA, Huntbach J (2006). Mass and shape as factors in intraocular foreign body injuries. Ophthalmology.

[7] Kong GY, Henderson RH, Sandhu SS, Essex RW, Allen PJ, Campbell WG (2015). Wound-related complications and clinical outcomes following open globe injury repair. Clin Exp Ophthalmol.

[8] Bhagat N, Nagori S, Zarbin M (2011). Post-traumatic infectious endophthalmitis. Survey of ophthalmology.

[9] Affeldt HW, Flynn, Forster RK, Mandelbaum S, Clarkson JG, Jarus GD (1987). Microbial endophthalmitis resulting from ocular trauma. Ophthalmology.

[10] Gokce G, Sobaci G, Ozgonul C (2015). Post-traumatic endophthalmitis: a mini-review. Semin Ophthalmol.

[11] Ferrari TM, Cardascia N, Di Gesu I (2001). Early versus late removal of retained intraocular foreign bodies. Retina.

[12] Jonas JB, Knorr HL, Budde WM (2000). Prognostic factors in ocular injuries caused by intraocular or retrobulbar foreign bodies. Ophthalmology.

[13] Colyer MH, Weber ED, Weichel ED, Dick JSB, Bower KS, Ward TP (2007). Delayed intraocular foreign body removal without endophthalmitis during operations iraqi freedom and enduring freedom. Ophthalmology.

[14] Soheilian M, Rafati N, Mohebbi M-R, Yazdani S, Habibabadi HF, Feghhi M (2007). Prophylaxis of acute post-traumatic bacterial endophthalmitis: a multicenter, randomized clinical trial of intraocular antibiotic injection, report 2. Arch Ophthalmol.

[15] Ariyasu RG, Kumar S, LaBree LD, Wagner DG, Smith RE (1995). Microor-ganisms cultured from the anterior chamber of ruptured globes at the time of repair. Am J Ophthalmol.

[16] Chhabra S, Kunimoto DY, Kazi L (2006). Endophthalmitis after open globe injury: microbiologic spectrum and susceptibilities of isolates. Am J Ophthalmol.

[17] Narang S, Gupta V, Gupta A (2003). Role of prophylactic intravitreal antibiotics in open globe injuries. Indian J Ophthalmol.

[18] Gariano RF, Kalina RE (1997). Posttraumatic fungal endophthalmitis resulting from Scopulariopsisbrevicaulis. Retina.

[19] Hammer ME, Harding S, Wynn P (1983). Post-traumatic fungal endophthalmitis caused by Exophialajeanselmei. Ann Ophthalmol.

[20] Ritesh Kumar Shah, Raghunand Byanju, Sangeeta Pradhan (2017). Management of posterior segment intraocular foreign body with vitrectomy: visual and anatomical outcome. Journal ofuniversal college of medical science.

[21] Ibraheem Waheed Ademola, Nazmum Naha, Ibraheem Anifat Boladale (2016). Clinical and demographic characteristics of intraocular foreign body Injury in a referral center: 3 YearsExperience. Pak J Ophthalmol.

[22] Öztaş Z, Nalçacı S, Afrashi F, Erakgün T, Menteş J, Değirmenci C (2015). Posterior segment intraocular foreign bodies: the effect of weight and size, early versus late vitrectomy and outcomes. UlusTravmaAcilCerrahiDerg.

